# Estimated Body Fat Percentage and Triglyceride‐Glucose Index for Identifying MASLD in Lean Asian Adults: A Cross‐Sectional Analysis

**DOI:** 10.1002/kjm2.70240

**Published:** 2026-05-26

**Authors:** Xiang‐Ran Kong, Ya‐Li Chen, Rui Li, Lu‐Xiang Shang, Sha Sha

**Affiliations:** ^1^ Central Hospital Affiliated to Shandong First Medical University Jinan China; ^2^ Department of Cardiology The Fourth People's Hospital of Jinan Jinan China; ^3^ Shandong Provincial Center for Disease Control and Prevention Jinan China; ^4^ Department of Health Management The First Affiliated Hospital of Shandong First Medical University & Shandong Provincial Qianfoshan Hospital Jinan China; ^5^ Department of Nephrology The First Affiliated Hospital of Shandong First Medical University & Shandong Provincial Qianfoshan Hospital Jinan China

**Keywords:** body fat percentage, Mendelian randomization, metabolic dysfunction‐associated steatotic liver disease, triglyceride‐glucose index

## Abstract

Metabolic dysfunction‐associated steatotic liver disease (MASLD) is increasingly prevalent among lean Asian populations, yet effective strategies for identifying high‐risk individuals remain limited. We investigated the associations of body fat percentage (BF%) and the triglyceride‐glucose (TyG) index with lean MASLD and evaluated their incremental diagnostic value in two independent studies (the NAGALA cohort and a Chinese health check‐up study). Lean MASLD was defined as imaging‐confirmed hepatic steatosis in individuals with BMI < 23 kg/m^2^. In both studies, participants with MASLD were older, more often male, and exhibited less favorable metabolic profiles. Multivariable analyses showed that the TyG index was consistently associated with increased odds of lean MASLD (adjusted OR per unit increase: 3.41 in NAGALA and 6.37 in the Chinese study), whereas associations of BF% varied by cohort and sex, with significant associations observed in NAGALA men and Chinese women (adjusted OR per unit increase: 1.20 and 1.24, respectively). In ROC analyses, the TyG index showed good discrimination (C‐statistics 0.778–0.875), and the addition of BF% further improved performance (0.805–0.901), corresponding to an absolute increase of approximately 0.02–0.05, with consistent improvements in net reclassification and discrimination (all *p* < 0.05). Mendelian randomization analyses supported a potential causal association between the TyG index and NAFLD, while no significant causal association was observed for BF%. Overall, BF% and the TyG index provide complementary information, and their combined use improves the identification of lean MASLD.

**Abbreviations**
ALT: alanine aminotransferase
AST: aspartate aminotransferase
AUC: area under the curve
BF%: body fat percentage
BMI: body mass index
CI: confidence Interval
DBP: diastolic blood pressure
FBG: fasting blood glucose
GGT: gamma‐glutamyl transferase
GWAS: genome‐wide association study
HDL: high‐density lipoprotein cholesterol
HR: hazard ratio
IDI: integrated discrimination improvement
IVW: inverse‐variance weighted
LDL: low‐density lipoprotein cholesterol
MASLD: metabolic dysfunction‐associated steatotic liver disease
MR: Mendelian randomization
MVMR: multivariable Mendelian randomization
NAFLD: non‐alcoholic fatty liver disease
NAGALA: NAfld in the Gifu area, longitudinal analysis
NRI: net reclassification improvement
OR: odds ratio
RCS: restricted cubic spline
ROC: receiver operating characteristic
SBP: systolic blood pressure
SNP: single nucleotide polymorphism
TC: total cholesterol
TG: triglycerides
TyG index: triglyceride‐glucose index
WC: waist circumference

## 1 Introduction

Metabolic dysfunction‐associated steatotic liver disease (MASLD), previously termed metabolic dysfunction‐associated fatty liver disease (MAFLD) or non‐alcoholic fatty liver disease (NAFLD), has become the most prevalent chronic liver disease worldwide and poses a major public health burden [1]. Although obesity is a well‐established risk factor, steatotic liver disease frequently occurs in individuals with normal body mass index (BMI), a phenotype known as lean MASLD [2, 3].

Lean MASLD is particularly common in Asian populations and is increasingly recognized as a clinically important condition [4]. Despite the absence of overt obesity, individuals with lean MASLD remain at increased risk of cardiovascular disease, liver‐related events, and all‐cause mortality [3, 5]. However, because lean individuals are often perceived as metabolically healthy, this condition is frequently under‐recognized in clinical practice, leading to missed opportunities for early detection and intervention [6]. This highlights the need for simple and practical tools to identify high‐risk individuals who may benefit from further imaging‐based evaluation.

Conventional screening indices for fatty liver disease, such as the fatty liver index and NAFLD fibrosis score, have shown suboptimal performance in lean populations [7, 8]. While more direct assessments of adiposity, including fat mass or visceral fat measurements, may better capture metabolic risk, they often require specialized equipment and are not suitable for large‐scale screening [9, 10]. Body fat percentage (BF%) provides a more direct estimate of adiposity than BMI and can be estimated using readily available clinical variables, making it a potentially useful alternative in routine settings [11, 12, 13].

Insulin resistance plays a central role in the pathogenesis of MASLD and is particularly relevant in lean individuals, in whom metabolic dysfunction may occur independently of obesity [2, 14]. The triglyceride‐glucose (TyG) index is a simple and validated surrogate marker of insulin resistance and has been consistently associated with MASLD across diverse populations [15, 16, 17].

Taken together, BF% and the TyG index reflect complementary aspects of metabolic dysfunction, namely adiposity and insulin resistance. However, their combined utility in identifying lean MASLD, particularly in comparison with conventional anthropometric measures such as BMI and waist circumference, remains insufficiently explored. Therefore, using data from two large health checkup‐based studies in Asian populations, this study aimed to evaluate the associations of estimated BF% and TyG index with lean MASLD, and compare their discriminative performance with traditional measures, complemented by Mendelian randomization (MR) analyses to assess potential causal relationships.

## 2 Materials and Methods

### 2.1 Study Design and Population

**FIGURE 1:**
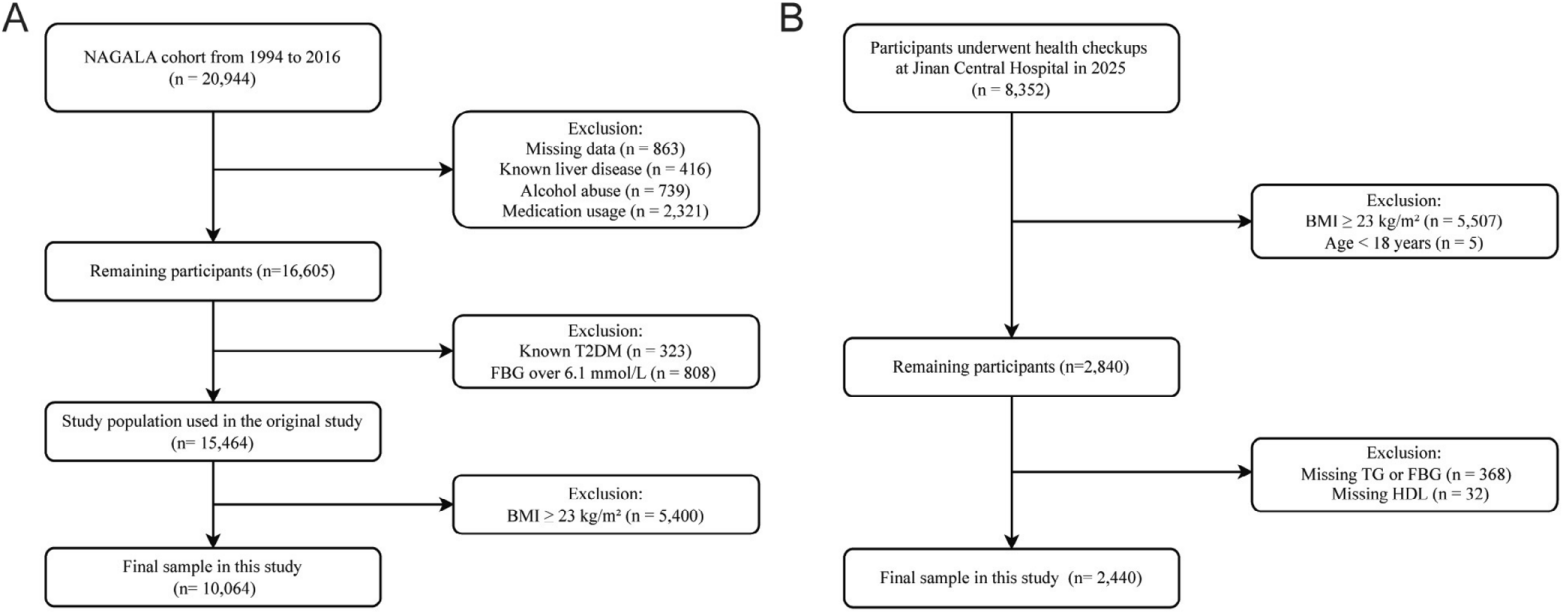
Flow diagram of study population selection for the NAGALA cohort (A) and Jinan health check‐up study (B).

This study was based on two cross‐sectional health check‐up datasets from Japan and China. The participant selection and exclusion processes for both studies are summarized in Figure 1. All analyses were performed on anonymized data. This study was conducted in accordance with the Strengthening the Reporting of Observational Studies in Epidemiology (STROBE) guidelines [18].

The Japanese dataset was derived from the NAfld in the Gifu Area, Longitudinal Analysis (NAGALA) cohort, an ongoing study based on routine health examinations conducted at Murakami Memorial Hospital, Japan [19, 20]. Between 1994 and 2016, a total of 20,944 individuals underwent periodic health check‐ups, with approximately 60% receiving clinical and laboratory assessments once or twice annually [21]. Baseline data were used for the present analysis. Participants with BMI < 23.0 kg/m^2^ were included, and those with missing data on BMI, triglycerides (TG), or fasting blood glucose (FBG) were excluded, resulting in 10,064 eligible participants. The NAGALA dataset is publicly available in the Dryad Digital Repository, and secondary analyses were conducted in accordance with the data use policy with appropriate citation of the original studies [22]. This study was approved by the Murakami Memorial Hospital ethics committee, and written informed consent was obtained from all participants.

The Chinese dataset was derived from routinely collected health examination data obtained in 2025 at the hospital affiliated with the first author. These data were retrospectively extracted from the institutional health check‐up database for the purpose of the present analysis. A total of 8352 individuals with available waist circumference measurements were initially screened. After excluding participants with BMI ≥ 23.0 kg/m^2^ and those with missing key variables, 2440 individuals were included in the final analysis. The study protocol was approved by the ethics committee of the first author's institution, which waived the requirement for informed consent due to the retrospective nature of the study and the use of de‐identified data.

### 2.2 Data Collection and Variable Definitions

Detailed information on data collection procedures for NAGALA cohort has been described previously in published literature [19, 23]. In brief, participants completed standardized questionnaires, underwent physical examinations, and received blood biochemical testing [19]. Demographic characteristics and lifestyle factors, including smoking, alcohol consumption, and physical activity, were assessed. For the Chinese dataset, data were extracted from the hospital information system. The collected variables included demographic characteristics, anthropometric measurements, blood pressure, and routine laboratory parameters. These variables were obtained as part of standard health examinations and were used for the present analysis.

The TyG index was calculated using the following formula: TyG = ln [TG (mg/dL) × FBG (mg/dL)/2] [24]. While, BF% was estimated using the CUN‐BAE equation, as follows: BF% = −44.988 + (0.503 × age) + (10.689 × sex) + (3.172 × BMI) − (0.026 × BMI^2^) + (0.181 × BMI × sex) − (0.02 × BMI × age) − (0.005 × BMI^2^ × sex) + (0.00021 × BMI^2^ × age), where sex is coded as 0 for males and 1 for females, and age is expressed in years [13].

### 2.3 MASLD Definition and Ascertainment

Hepatic steatosis was assessed using abdominal ultrasonography performed by trained technicians as part of routine health examinations. The diagnosis of hepatic steatosis was based on standard ultrasound criteria in accordance with established clinical guidelines and previous studies [19, 25].

According to current consensus criteria [26], MASLD was defined as the presence of hepatic steatosis in combination with at least one cardiometabolic risk factor (CMRF). In both datasets, CMRFs were determined based on available variables and included the following components: (1) abdominal obesity, defined as waist circumference (WC) ≥ 90 cm in men or ≥ 80 cm in women; (2) impaired glucose metabolism, defined as FBG ≥ 5.6 mmol/L or hemoglobin A1c (HbA1c) ≥ 5.7%; (3) elevated blood pressure, defined as systolic blood pressure (SBP) ≥ 130 mmHg or diastolic blood pressure (DBP) ≥ 85 mmHg; (4) hypertriglyceridemia, defined as TG ≥ 1.70 mmol/L; and (5) reduced high‐density lipoprotein (HDL) cholesterol, defined as HDL cholesterol ≤ 1.0 mmol/L in men or ≤ 1.3 mmol/L in women. Lean MASLD was defined as MASLD occurring in individuals with BMI < 23 kg/m^2^ [27].

### 2.4 Covariates

Both studies were adjusted for age, sex, SBP, DBP, WC, alanine aminotransferase (ALT), aspartate aminotransferase (AST), gamma‐glutamyl transferase (GGT), total cholesterol (TC), and HDL. Sex was treated as a categorical variable, while all other covariates were analyzed as continuous variables. In addition, the NAGALA cohort was further adjusted for smoking status, alcohol consumption, and physical activity, while the Chinese dataset was additionally adjusted for low‐density lipoprotein (LDL).

Because BF% was estimated using a formula incorporating age and sex, these variables were not included as covariates in BF%‐MASLD models. To account for potential sex‐specific effects, analyses involving BF% were conducted using sex‐stratified models.

### 2.5 Statistical Analysis

Data were analyzed using *SPSS* version 25.0 and *R* version 3.5.2. Continuous variables were expressed as mean ± standard deviation (SD) or median (interquartile range, IQR), and categorical variables as frequencies and percentages. Group comparisons were performed using Student's *t*‐test or the Mann–Whitney *U* test for continuous variables, and the chi‐square test for categorical variables, as appropriate.

Univariate and multivariate logistic regression models were used to estimate odds ratios (ORs) and 95% confidence intervals (CIs) for the associations of BF% and the TyG index (analyzed as both quartiles and continuous variables) with lean MASLD. Restricted cubic spline (RCS) models with three knots were applied to assess potential nonlinear relationships. Subgroup analyses stratified by age (< 60 and ≥ 60 years) were conducted to evaluate potential effect modification. Sensitivity analyses were performed using imaging‐defined hepatic steatosis alone, without requiring cardiometabolic risk factors, to assess the robustness of the findings.

Receiver operating characteristic (ROC) curve analysis was used to evaluate the discriminative performance of BF%, the TyG index, and their combination (BF% + TyG), with calculation of the area under the curve (AUC). Differences in AUCs were compared using the DeLong test. The incremental predictive value of combining BF% with TyG index was further assessed using the net reclassification improvement (NRI) and integrated discrimination improvement (IDI) [28]. A two‐sided *p*‐value < 0.05 was considered statistically significant.

### 2.6 Two‐Sample MR Analysis

We conducted a two‐sample MR analysis to investigate the potential causal effects of genetically predicted BF% and TyG index on NAFLD, following the *STROBE‐MR* guidelines [29]. Genetic instruments for BF% were obtained from the GWAS Catalog, including a meta‐analysis of 100,716 participants [30], and a UK Biobank study of 174,488 individuals [31]. Genetic variants for TyG index were derived from a GWAS of 273,368 UK Biobank participants without diabetes or lipid disorders [32]. Summary statistics for NAFLD were obtained from the FinnGen consortium (round 12) [33]. A detailed description of all data sources is provided in Table S1. All original studies received ethical approval, and informed consent was obtained from all participants.

Single nucleotide polymorphisms (SNPs) associated with exposures were selected using a genome‐wide significance threshold (*p* < 5 × 10^−8^). Linkage disequilibrium clumping was applied to ensure independence of instruments (*R* ^2^ < 0.001 within a 10,000 kb window). Alleles were harmonized across datasets, and palindromic SNPs were excluded to minimize strand ambiguity. The inverse variance weighted (IVW) method was used as the primary MR approach. Sensitivity analyses were conducted to assess heterogeneity and potential horizontal pleiotropy. In addition, multivariable MR (MVMR) was performed to evaluate the independent effects of BF% and the TyG index on NAFLD. All MR analyses were implemented using the TwoSampleMR, MR‐PRESSO, and MVMR *R* packages.

## 3 Results

### 3.1 Study Population Characteristics

Characteristics of participants stratified by MASLD status in the two studies are summarized in Tables 1 and 2. In the NAGALA cohort and the Chinese health check‐up dataset, 353 (3.12%) and 249 (10.2%) participants were classified as having MASLD, respectively. Across both studies, participants with MASLD were consistently older and more likely to be male compared with those without MASLD. In addition, individuals with MASLD exhibited a less favorable metabolic profile, including higher levels of BMI, WC, blood pressure, liver enzymes, glucose, and lipid parameters.

**TABLE 1:** Characteristics of participants with and without MASLD in the NAGALA cohort.

Characteristics	Individuals without MASLD (*n* = 9711)	Individuals with MASLD (*n* = 353)	*p*
Age	43.07 ± 8.99	46.29 ± 8.46	< 0.001
Sex			< 0.001
Female	5537 (57.02)	74 (20.96)	
Male	4174 (42.98)	279 (79.04)	
Alcohol consumption			0.898
Non	7663 (78.91)	281 (79.60)	
Light	1041 (10.72)	34 (9.63)	
Moderate	741 (7.63)	29 (8.22)	
Heavy	266 (2.74)	9 (2.55)	
Smoking			< 0.001
Never	6345 (65.34)	171 (48.44)	
Past	1510 (15.55)	85 (24.08)	
Current	1856 (19.11)	97 (27.48)	
Regular exercise	1780 (18.33)	47 (13.31)	0.016
BMI, kg/m^2^	20.26 ± 1.70	21.80 ± 0.96	< 0.001
WC, cm	71.62 ± 6.35	78.70 ± 4.68	< 0.001
BF%	23.47 ± 6.16	22.09 ± 5.37	< 0.001
SBP, mmHg	110.06 ± 13.35	120.01 ± 14.54	< 0.001
DBP, mmHg	68.50 ± 9.44	75.60 ± 10.05	< 0.001
ALT, U/L	15 (12, 19)	23 (18, 32)	< 0.001
AST, U/L	17 (14, 20)	19 (16, 24)	< 0.001
GGT, U/L	13 (10, 18)	22 (16, 33)	< 0.001
TC, mmol/L	5.00 ± 0.84	5.45 ± 0.88	< 0.001
TG, mmol/L	0.72 ± 0.47	1.43 ± 0.91	< 0.001
HDL, mmol/L	1.58 ± 0.40	1.22 ± 0.37	< 0.001
HbA1c, %	5.14 ± 0.31	5.33 ± 0.36	< 0.001
FBG, mmol/L	5.06 ± 0.40	5.46 ± 0.39	< 0.001
TyG	7.82 ± 0.57	8.57 ± 0.57	< 0.001

Abbreviations: ALT, alanine aminotransferase; AST, aspartate aminotransferase; BF%, body fat percentage; DBP, diastolic blood pressure; FBG, fasting blood glucose; GGT, gamma‐glutamyl transferase; HbA1c, glycated hemoglobin; HDL, high‐density lipoprotein cholesterol; MASLD, metabolic dysfunction–associated steatotic liver disease; SBP, systolic blood pressure; TC, total cholesterol; TG, triglycerides; TyG, triglyceride‐glucose index; WC, waist circumference.

**TABLE 2:** Characteristics of participants with and without MASLD in Jinan health check‐up study.

Characteristics	Individuals without MASLD (*n* = 2191)	Individuals with MASLD (*n* = 249)	*p*
Age	39.66 ± 12.69	50.59 ± 13.80	< 0.001
Sex			< 0.001
Female	1697 (77.5%)	162 (65.1%)	
Male	494 (22.5%)	87 (34.9%)	
BMI, kg/m^2^	20.78 ± 1.55	21.89 ± 0.94	< 0.001
WC, cm	75.49 ± 7.01	82.40 ± 6.90	< 0.001
BF%	26.19 ± 6.09	28.34 ± 6.91	< 0.001
SBP, mmHg	118.85 ± 14.57	131.09 ± 17.16	< 0.001
DBP, mmHg	71.63 ± 9.50	77.58 ± 10.44	< 0.001
ALT, U/L	12.10 (9.30, 16.40)	16.90 (12.30, 24.27)	< 0.001
AST, U/L	16.50 (14.20, 19.50)	18.30 (15.90, 22.20)	< 0.001
GGT, U/L	12.80 (10.00, 17.00)	19.80 (13.20, 31.00)	< 0.001
TC, mmol/L	4.72 ± 0.89	5.07 ± 1.03	< 0.001
TG, mmol/L	0.90 ± 0.43	1.66 ± 0.91	< 0.001
LDL, mmol/L	2.32 ± 0.63	2.76 ± 0.75	< 0.001
HDL, mmol/L	1.53 ± 0.28	1.36 ± 0.29	< 0.001
FBG, mmol/L	4.83 ± 0.67	5.52 ± 1.32	< 0.001
TyG	8.06 ± 0.43	8.76 ± 0.53	< 0.001

Abbreviations: ALT, alanine aminotransferase; AST, aspartate aminotransferase; BF%, body fat percentage; BMI, body mass index; DBP, diastolic blood pressure; FBG, fasting blood glucose; GGT, gamma‐glutamyl transferase; HDL, high‐density lipoprotein cholesterol; LDL, low‐density lipoprotein cholesterol; MASLD, metabolic dysfunction‐associated steatotic liver disease; SBP, systolic blood pressure; TC, total cholesterol; TG, triglycerides; TyG, triglyceride‐glucose index; WC, waist circumference.

### 3.2 Associations of BF% and TyG Index With Lean MASLD

Multivariable logistic regression analyses showed that the TyG index was consistently associated with increased odds of lean MASLD across both studies, whereas the associations of BF% varied by cohort and sex (Table 3). In the NAGALA cohort, higher BF% was associated with increased odds of lean MASLD in men, with significant associations observed in the higher quartiles and for BF% as a continuous variable. Among women in the same cohort, the association was weaker and not consistently significant after adjustment. In the Chinese health check‐up dataset, BF% was not independently associated with lean MASLD in men after multivariable adjustment, whereas in women, the highest BF% quartile and BF% as a continuous variable remained significantly associated with lean MASLD.

**TABLE 3:** Associations of BF% and TyG with lean MASLD across the two studies using logistic regression models (OR and 95% CI).

	NAGALA cohort			Jinan health check‐up study		
	Case/*N*	Unadjusted model	Adjusted model	Case/*N*	Unadjusted model	Adjusted model
BF% (male)						
T1	12/1114	Reference	Reference	4/146	Reference	Reference
T2	39/1113	3.33 (1.79–6.68)	1.39 (0.71–2.90)	18/145	5.03 (1.82–17.77)	2.32 (0.76–8.73)
T3	97/1113	8.77 (4.98–16.92)	2.43 (1.27–5.02)	31/145	9.65 (3.69–33.16)	2.39 (0.78–9.06)
T4	131/1113	12.25 (7.03–23.48)	2.89 (1.38–6.43)	34/145	10.87 (4.18–37.23)	2.57 (0.62–12.26)
BF% continuous (male)	—	1.32 (1.26–1.39)	1.20 (1.08–1.34)	—	1.21 (1.13–1.31)	1.11 (0.93–1.34)
BF% (female)						
T1	0/1403	—	—	3/465	Reference	Reference
T2	4/1403	Reference	Reference	15/465	5.13 (1.68–22.27)	2.28 (0.72–10.10)
T3	18/1403	4.55 (1.69–15.77)	2.47 (0.85–9.31)	31/465	11.00 (3.89–46.06)	2.58 (0.85–11.23)
T4	52/1402	13.47 (5.50–44.63)	2.86 (0.96–10.94)	113/464	49.58 (18.51–202.48)	4.86 (1.47–22.26)
BF% continuous (female)	—	1.53 (1.40–1.69)	1.16 (1.00–1.35)	—	1.49 (1.40–1.59)	1.24 (1.09–1.43)
TyG						
T1	8/2516	Reference	Reference	6/610	Reference	Reference
T2	26/2516	3.27 (1.55–7.75)	1.84 (0.85–4.47)	14/610	2.36 (0.94–6.72)	1.39 (0.54–4.00)
T3	51/2516	6.49 (3.25–14.81)	2.08 (0.99–4.93)	40/610	7.06 (3.20–18.67)	2.60 (1.13–7.07)
T4	268/2516	37.37 (19.77–82.60)	5.33 (2.60–12.52)	189/610	45.19 (21.69–115.63)	8.70 (3.87–23.37)
TyG continuous	—	8.41 (6.94–10.24)	3.41 (2.60–4.49)	—	17.61 (12.78–24.66)	6.37 (4.29–9.59)

Abbreviations: BF%, body fat percentage; CI, confidence interval; MASLD, metabolic dysfunction‐associated steatotic liver disease; OR, odds ratio; TyG, triglyceride‐glucose index.

**FIGURE 2:**
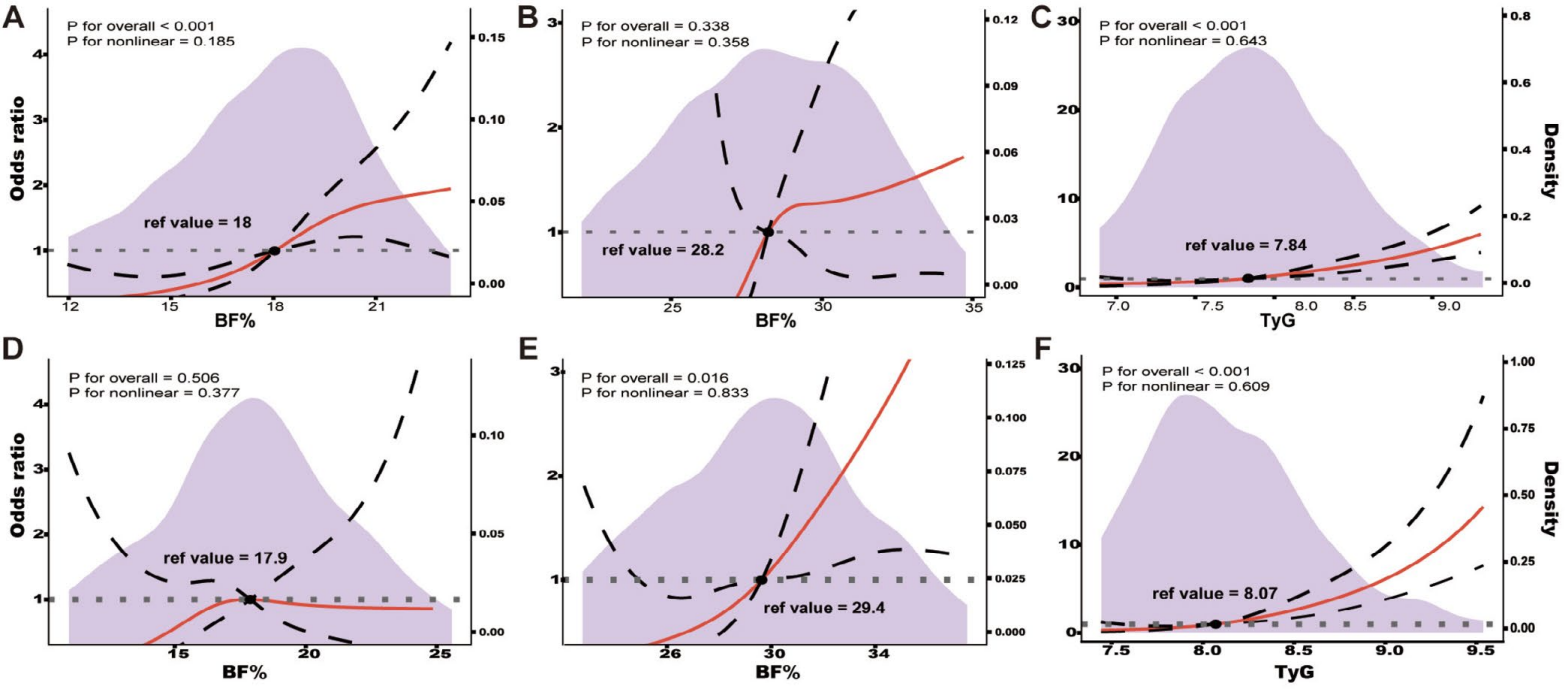
RCS analyses of the associations between BF%, the TyG index, and lean MASLD. Panels A–C present results from the NAGALA cohort, showing the associations of BF% with lean MASLD in males (A) and females (B), and the association of the TyG index in the overall population (C). Panels D–F present corresponding analyses from the Chinese health check‐up dataset, including BF% in males (D) and females (E), and the TyG index (F).

Restricted cubic spline analyses further supported these findings (Figure 2). The TyG index showed clear positive dose–response relationships with lean MASLD in both cohorts, with significant overall associations and no evidence of marked nonlinearity. For BF%, the overall association was significant in NAGALA men and in women from the Chinese dataset, but not in NAGALA women or Chinese men. In all BF% models, there was no strong evidence of nonlinear association.

Sensitivity analyses using imaging‐defined hepatic steatosis alone yielded broadly similar results, supporting the robustness of the main findings (Tables S2 and S3). Subgroup analyses stratified by age showed generally similar patterns across strata, without consistent evidence of interaction (Table S4).

### 3.3 Incremental Diagnostic Value of BF% and TyG Index for Lean MASLD

ROC analyses showed that the TyG index demonstrated good discriminative ability for lean MASLD across both cohorts, and the addition of BF% further improved model performance in all sex‐specific analyses (Table 4). The C‐statistic increased from 0.778 to 0.805 in NAGALA males and from 0.794 to 0.818 in Chinese males, and from 0.831 to 0.878 and from 0.875 to 0.901 among females, respectively. These improvements were consistently supported by significant NRI and IDI values across all analyses.

**TABLE 4:** Incremental diagnostic value of combining BF% with the TyG index for identifying lean MAFLD.

Study	Indicator	C‐statistic	*p*	NRI	*p*	IDI	*p*
NAGALA cohort (male)	TyG index	0.778 (0.749–0.806)	< 0.001	Reference		Reference	
	BF%	0.710 (0.683–0.736)	< 0.001	—		—	
	BF% + TyG	0.805 (0.780–0.830)	< 0.001	0.542 (0.420–0.647)	< 0.001	0.017 (0.011–0.022)	< 0.001
NAGALA cohort (female)	TyG index	0.831 (0.785–0.878)	< 0.001	Reference		Reference	
	BF%	0.824 (0.785–0.862)	< 0.001	—		—	
	BF% + TyG	0.878 (0.842–0.914)	< 0.001	0.685 (0.469–0.908)	< 0.001	0.019 (0.009–0.030)	< 0.001
Jinan health check‐up study (male)	TyG index	0.794 (0.737–0.851)	< 0.001	Reference		Reference	
	BF%	0.689 (0.637–0.740)	< 0.001	—		—	
	BF% + TyG	0.818 (0.766–0.870)	< 0.001	0.371 (0.147–0.599)	< 0.001	0.018 (0.003–0.036)	0.020
Jinan health check‐up study (female)	TyG index	0.875 (0.849–0.901)	< 0.001	Reference		Reference	
	BF%	0.824 (0.793–0.854)	< 0.001	—		—	
	BF% + TyG	0.901 (0.879–0.923)	< 0.001	0.563 (0.404–0.719)	< 0.001	0.046 (0.030–0.064)	< 0.001

Abbreviations: BF%, body fat percentage; IDI, integrated discrimination improvement; MAFLD, metabolic dysfunction‐associated steatotic liver disease; NRI, net reclassification improvement; TyG, triglyceride‐glucose index.

**FIGURE 3:**
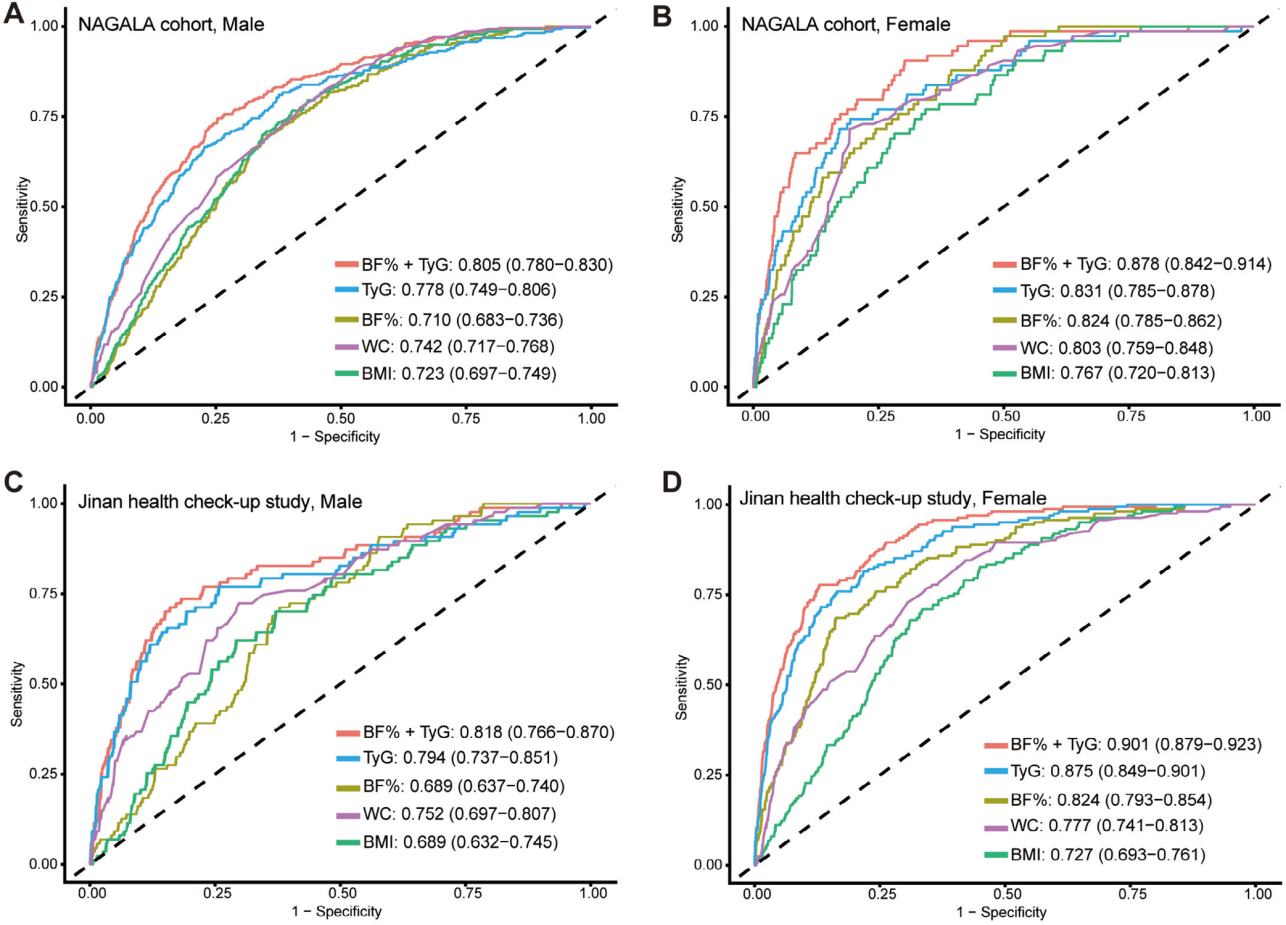
ROC curves for the identification of lean MASLD using BF%, the TyG index, and conventional anthropometric measures. Panels A and B show results from the NAGALA cohort for males (A) and females (B), respectively. Panels C and D present corresponding results from the Chinese health check‐up dataset for males (C) and females (D).

In comparisons with conventional anthropometric indices, BF% showed discriminative ability comparable to or better than BMI in all analyses, with no significant difference observed in males in either study (see Figure 3). Compared with WC, the performance of BF% was generally similar, although differences varied across subgroups, with some analyses showing superior performance of BF% and others showing comparable or slightly lower discrimination.

### 3.4 Two‐Sample MR Analysis

To assess the causal relationships of BF% and the TyG index with NAFLD, we performed both univariable and multivariable MR analyses. As shown in Table 5, the TyG index was significantly associated with an increased risk of NAFLD (OR = 1.738, 95% CI: 1.158–2.609, *p* = 0.008). In contrast, BF% was not associated with NAFLD in either data source (meta‐analysis: OR = 1.455, *p* = 0.563; UK Biobank: OR = 1.393, *p* = 0.107). In multivariable MR analyses adjusting for both exposures, the association between the TyG index and NAFLD remained robust (meta‐analysis: OR = 2.288, *p* = 0.006; UK Biobank: OR = 2.664, *p* = 0.011), suggesting an independent causal effect of insulin resistance‐related metabolic dysfunction on NAFLD risk.

**TABLE 5:** Associations of genetically predicted BF% and TyG index with NAFLD.

Exposure	Method	nSNP	*p*	OR (95% CI)	*p* value for heterogeneity *Q* test	*p* value for egger intercept
BF% from meta	IVW	9	0.563	1.455 (0.408–5.184)	3.63 × 10^−7^	0.265
BF% from UKB	IVW	79	0.107	1.393 (0.931–2.084)	8.99 × 10^−5^	0.217
TyG index	IVW	99	0.008	1.738 (1.158–2.609)	3.99 × 10^−6^	0.125
MVMR						
BF% from meta	IVW	47	0.538	0.810 (0.414–1.585)	2.36 × 10^−6^	0.389
TyG index	IVW	47	0.006	2.288 (1.272–4.116)		
MVMR						
BF% from UKB	IVW	28	0.104	0.134 (0.012–1.508)	0.001	0.883
TyG index	IVW	28	0.011	2.664 (1.256–5.651)		

Abbreviations: BF%, body fat percentage; CI, confidence interval; IVW, inverse‐variance weighted; MVMR, multivariable Mendelian randomization; NAFLD, nonalcoholic fatty liver disease; nSNP, number of single nucleotide polymorphisms; OR, odds ratio; TyG, triglyceride‐glucose index; UKB, UK Biobank.

## 4 Discussion

This study provides consistent evidence from two independent Asian studies that both BF% and the TyG index are associated with lean MASLD. Importantly, we demonstrate that combining these two indices improves the identification of high‐risk individuals beyond either metric alone. In addition, MR analyses support a potential causal role of the TyG index in NAFLD development. Together, these findings suggest that integrating body composition and metabolic indicators offers a more informative approach for identifying lean individuals at risk of MASLD.

Lean MASLD is increasingly recognized as a clinically relevant phenotype, particularly in Asian populations, where metabolic abnormalities often occur at lower BMI levels [34]. In this context, individuals with normal BMI may still harbor excess adiposity and metabolic dysfunction, leading to under‐recognition in routine clinical practice. Our findings highlight that BF%, as an indicator of body fat composition, captures aspects of adiposity that are not reflected by BMI, while the TyG index reflects insulin resistance‐related metabolic dysfunction. The combined use of these measures therefore provides complementary information, enabling improved detection of metabolic heterogeneity among lean individuals [35].

The association between BF% and lean MASLD showed heterogeneity across cohorts and sexes, with significant associations observed in NAGALA men and Chinese women but not consistently across all subgroups. This pattern may reflect differences in fat distribution, hormonal influences, and population characteristics, further underscoring the complexity of lean MASLD [36]. In contrast, the TyG index showed more consistent associations across analyses, suggesting that insulin resistance may represent a common metabolic pathway underlying lean MASLD.

Notably, our MR analyses supported a potential causal association between the TyG index and NAFLD, whereas no significant causal evidence was observed for BF%. This discrepancy may partly reflect limitations of currently available GWAS datasets, which are predominantly derived from European populations and lack stratification by BMI [37]. Consequently, these datasets may be insufficiently sensitive to detect genetic determinants of adiposity‐related risk specifically in lean individuals, particularly in Asian populations, who tend to exhibit greater metabolic vulnerability at lower BMI levels [38]. These findings underscore the need for future GWAS focusing on lean MASLD phenotypes, especially in non‐European populations.

From a mechanistic perspective, our findings are supported by established biological links between adiposity, insulin resistance, and hepatic steatosis. Excess body fat is associated with increased free fatty acid flux, dysregulated lipid metabolism, and compensatory hyperinsulinemia, all of which contribute to hepatic lipid accumulation [39]. From a clinical and public health perspective, our results have practical implications. Both BF% and the TyG index can be derived from routinely available data without the need for specialized imaging or invasive procedures. Their combined use improved discrimination, reclassification, and overall diagnostic performance, supporting their potential utility as simple screening tools to identify lean individuals who may benefit from further evaluation, such as hepatic imaging. This is particularly relevant in resource‐limited settings, where widespread imaging‐based screening is not feasible.

## 5 Limitations

Several limitations should be considered. First, this study was based on secondary analyses of existing datasets, and variable availability was limited. In particular, information on medication usage and clinical management was unavailable in the NAGALA dataset, which may have introduced residual confounding. In addition, exclusion criteria applied in the original studies may have affected the representativeness of the study population, potentially limiting generalizability. Second, hepatic steatosis was assessed using ultrasonography, which has limited sensitivity for detecting mild steatosis and does not provide information on fibrosis or steatohepatitis. Therefore, the clinical significance of early or subclinical disease may not be fully captured. Third, the TyG index is influenced by fasting triglyceride and glucose levels, which may vary depending on dietary intake and fasting conditions, and the use of single measurements may introduce measurement variability. Fourth, BF% in this study was estimated using prediction equations rather than direct measurement methods, which may introduce estimation error. Fifth, the cross‐sectional nature of the observational analyses limits causal inference and raises the possibility of reverse causation, particularly in mediation analyses. Finally, MR analyses were constrained by the availability of GWAS data, which were primarily derived from European populations and not specific to lean MASLD, and we were unable to perform mediation or bidirectional MR analyses due to limited summary statistics.

## 6 Conclusion

In conclusion, our findings suggest that BF% and the TyG index provide complementary information for identifying lean MASLD in Asian populations, and their combined use may improve early detection and risk stratification in clinical practice.

## Funding

This work was supported by the Shandong Provincial Natural Science Foundation (ZR2021QH086) and the Shandong Postdoctoral Science Foundation (SDCX‐ZG‐202400007).

## Ethics Statement

This study was conducted in accordance with the Declaration of Helsinki. All original studies were approved by their respective institutional ethics committees. Written informed consent was obtained from participants where applicable, while the requirement for informed consent was waived for the Chinese health check‐up dataset due to its retrospective use of anonymized data.

## Conflicts of Interest

The authors declare no conflicts of interest.

## Supporting information


**Table S1:** Overview of GWAS data sources.
**Table S2:** Sensitivity analysis of the associations between BF% and TyG index with lean NAFLD in the NAGALA cohort.
**Table S3:** Sensitivity analysis of the associations between BF% and TyG index with lean NAFLD in the Jinan health check‐up study.
**Table S4:** Subgroup analysis of the associations between BF% and TyG index and lean MASLD by age groups in the two cohorts.

## Data Availability

The data that support the findings of this study are available from the corresponding author upon reasonable request.
