# Supplementary material for: Estimated Body Fat Percentage and Triglyceride‐Glucose Index for Identifying MASLD in Lean Asian Adults: A Cross‐Sectional Analysis
Source: Kaohsiung J Med Sci. 2026 May 26:e70240. Online ahead of print. doi: 10.1002/kjm2.70240 (PMC13399757; doi:10.1002/kjm2.70240)
Supplement: Supplementary file 1 — Table S1: Overview of GWAS data sources. Table S2: Sensitivity analysis of the associations between BF% and TyG index with lean NAFLD in the NAGALA cohort. Table S3: Sensitivity analysis of the associations between BF% and TyG index with lean NAFLD in the Jinan health check‐up study. Table S4: Subgroup analysis of the associations between BF% and TyG index and lean MASLD by age groups in the two cohorts. [file KJM2-9999-e70240-s001.docx]

**Estimated body fat percentage and triglyceride-glucose index for identifying MASLD in lean Asian adults: A cross-sectional analysis**

**Supplementary files:**

Supplementary Table S1. Overview of GWAS data sources.

Supplementary Table S2. Sensitivity analysis of the associations between BF% and TyG index with lean NAFLD in the NAGALA cohort.

Supplementary Table S3. Sensitivity analysis of the associations between BF% and TyG index with lean NAFLD in the Jinan health check-up study.

Supplementary Table S4. Subgroup analysis of the associations between BF% and TyG index and lean MASLD by age groups in the two studies.

**Supplementary Table S1. Overview of GWAS data sources.**

| Trait | Case/control | Sample size | Consortium | Ancestry | GWAS ID | PMID/Link |
| --- | --- | --- | --- | --- | --- | --- |
| Exposure |  |  |  |  |  |  |
| BF% | – | 100,716 | GWAS meta-analysis | 88.7% of European | GCST003435 | 26833246 |
| BF% | – | 174,488 | UK biobank | European | GCST90309831 | 38351177 |
| TyG | – | 273,368 | UK biobank | European | – | 33553250 |
| Outcome |  |  |  |  |  |  |
| NAFLD | 3,504/496,884 | 500,388 | FinnGen | European | – | https://r12.finngen.fi/ |

Abbreviations: BF%, Body fat percentage; TyG, Triglyceride-glucose index; NAFLD, Nonalcoholic fatty liver disease; GWAS, Genome-wide association study; PMID, PubMed Identifier.

**Supplementary Table S2. Sensitivity analysis of the associations between BF% and TyG index with lean NAFLD in the NAGALA cohort.**

|  | Case/N | Unadjusted Model | *P* value |  | Adjusted model | *P* value |  |
| --- | --- | --- | --- | --- | --- | --- | --- |
| BF% (male) |  |  |  |  |  |  |  |
| T1 | 25/1,114 | Reference | - |  | Reference | - |  |
| T2 | 71/1,113 | 2.97 (1.89 - 4.81) | <0.001 |  | 1.43 (0.87 - 2.41) | 0.165 |  |
| T3 | 157/1,113 | 7.15 (4.74 - 11.26) | <0.001 |  | 2.51 (1.54 - 4.23) | <0.001 |  |
| T4 | 181/1,113 | 8.46 (5.63 - 13.27) | <0.001 |  | 2.72 (1.54 - 4.94) | <0.001 |  |
| BF% continuous (male) | - | 1.28 (1.23 - 1.33) | <0.001 |  | 1.20 (1.10 - 1.30) | <0.001 |  |
| BF% (female) |  |  |  |  |  |  |  |
| T1 | 1/1,403 | Reference | - |  | Reference | - |  |
| T2 | 5/1,403 | 5.01 (0.81 - 96.13) | 0.141 |  | 2.88 (0.44 - 56.30) | 0.342 |  |
| T3 | 30/1,403 | 30.63 (6.56 - 545.98) | <0.001 |  | 11.20 (2.24 - 204.83) | 0.020 |  |
| T4 | 78/1,402 | 82.60 (18.32 - 1458.09) | <0.001 |  | 14.99 (2.88 - 277.46) | 0.010 |  |
| BF% continuous (female) | - | 1.50 (1.39 - 1.62) | <0.001 |  | 1.22 (1.09 - 1.38) | <0.001 |  |
| TyG |  |  |  |  |  |  |  |
| T1 | 23/2,516 | Reference | - |  | Reference | - |  |
| T2 | 51/2,516 | 2.24 (1.38 - 3.75) | 0.001 |  | 1.38 (0.83 - 2.37) | 0.225 |  |
| T3 | 113/2,516 | 5.10 (3.31 - 8.20) | <0.001 |  | 2.06 (1.28 - 3.45) | 0.004 |  |
| T4 | 361/2,516 | 18.16 (12.15 - 28.57) | <0.001 |  | 3.85 (2.39 - 6.47) | <0.001 |  |
| TyG continuous | - | 5.82 (4.99 - 6.82) | <0.001 |  | 2.58 (2.07 - 3.23) | <0.001 |  |

Abbreviations: BF%, body fat percentage; TyG, triglyceride-glucose index; NAFLD, non-alcoholic fatty liver disease; OR, odds ratio; HR, hazard ratio; CI, confidence interval.

**Supplementary Table S3. Sensitivity analysis of the associations between BF% and TyG index with lean NAFLD in the Jinan health check-up study.**

|  | Case/N | Unadjusted Model | *P* value |  | Adjusted model | *P* value |  |
| --- | --- | --- | --- | --- | --- | --- | --- |
| BF% (male) |  |  |  |  |  |  |  |
| T1 | 9/146 | Reference | - |  | Reference | - |  |
| T2 | 23/145 | 2.87 (1.32 - 6.77) | 0.011 |  | 1.44 (0.61 - 3.63) | 0.419 |  |
| T3 | 38/145 | 5.41 (2.61 - 12.36) | <0.001 |  | 1.71 (0.71 - 4.40) | 0.242 |  |
| T4 | 37/145 | 5.22 (2.51 - 11.94) | <0.001 |  | 1.85 (0.56 - 6.30) | 0.319 |  |
| BF% continuous (male) | - | 1.17 (1.10 - 1.25) | <0.001 |  | 1.11 (0.96 - 1.31) | 0.178 |  |
| BF% (female) |  |  |  |  |  |  |  |
| T1 | 7/465 | Reference | - |  | Reference | - |  |
| T2 | 23/465 | 3.40 (1.52 - 8.66) | 0.005 |  | 1.90 (0.82 - 4.92) | 0.153 |  |
| T3 | 37/465 | 5.66 (2.65 - 13.98) | <0.001 |  | 1.96 (0.86 - 5.06) | 0.131 |  |
| T4 | 124/464 | 23.86 (11.84 - 56.99) | <0.001 |  | 4.36 (1.74 - 12.13) | 0.003 |  |
| BF% continuous (female) | - | 1.42 (1.35 - 1.51) | <0.001 |  | 1.29 (1.16 - 1.46) | <0.001 |  |
| TyG |  |  |  |  |  |  |  |
| T1 | 15/610 | Reference | - |  | Reference | - |  |
| T2 | 24/610 | 1.62 (0.85 - 3.19) | 0.147 |  | 1.07 (0.55 - 2.14) | 0.839 |  |
| T3 | 62/610 | 4.49 (2.59 - 8.27) | <0.001 |  | 2.06 (1.15 - 3.91) | 0.02 |  |
| T4 | 197/610 | 18.92 (11.41 - 33.83) | <0.001 |  | 4.74 (2.63 - 9.03) | <0.001 |  |
| TyG continuous | - | 11.00 (8.33 - 14.70) | <0.001 |  | 4.51 (3.17 - 6.47) | <0.001 |  |

Abbreviations: BF%, body fat percentage; TyG, triglyceride-glucose index; NAFLD, non-alcoholic fatty liver disease; OR, odds ratio; HR, hazard ratio; CI, confidence interval.

**Supplementary Table S4. Subgroup analysis of the associations between BF% and TyG index and lean MASLD by age groups in the two cohorts.**

| Study | Subgroup | *OR* (95%CI) | *P* value | *P* for interaction |
| --- | --- | --- | --- | --- |
| NAGALA cohort (male) |  |  |  |  |
| BF% continuous | Age < 60 | 1.18 (1.08 - 1.28) | <0.001 | 0.995 |
|  | Age ≥ 60 | 1.43 (0.86 - 2.39) | 0.170 |  |
| TyG continuous | Age < 60 | 3.38 (2.43 - 4.70) | <0.001 | 0.056 |
|  | Age ≥ 60 | 1.16 (0.30 - 4.57) | 0.828 |  |
| NAGALA cohort (female) |  |  |  |  |
| BF% continuous | Age < 60 | 1.28 (1.12 - 1.46) | <0.001 | 0.331 |
|  | Age ≥ 60 | NE | <0.001 |  |
| TyG continuous | Age < 60 | 3.86 (2.14 - 6.94) | <0.001 | 0.143 |
|  | Age ≥ 60 | NE | 0.998 |  |
| Jinan Check-up study (male) |  |  |  |  |
| BF% continuous | Age < 60 | 1.26 (1.21 - 1.32) | <0.001 | 0.053 |
|  | Age ≥ 60 | 1.41 (1.28 - 1.55) | <0.001 |  |
| TyG continuous | Age < 60 | 5.01 (4.02 - 6.25) | <0.001 | 0.065 |
|  | Age ≥ 60 | 4.34 (2.92 - 6.45) | <0.001 |  |
| Jinan Check-up study (female) |  |  |  |  |
| BF% continuous | Age < 60 | 1.30 (1.25 - 1.35) | <0.001 | 0.985 |
|  | Age ≥ 60 | 1.37 (1.14 - 1.65) | <0.001 |  |
| TyG continuous | Age < 60 | 4.46 (3.66 - 5.43) | <0.001 | 0.132 |
|  | Age ≥ 60 | 4.28 (2.27 - 8.05) | <0.001 |  |

Abbreviations: BF%, body fat percentage; TyG, triglyceride-glucose index; MASLD, metabolic dysfunction-associated steatotic liver disease; OR, odds ratio; HR, hazard ratio; CI, confidence interval; NE, not estimable.
